# Analysis of Pro-Inflammatory and Anti-Inflammatory Cytokine Serum Concentrations in Pediatric Patients with Neuroblastoma: A Preliminary Study

**DOI:** 10.3390/biomedicines13071517

**Published:** 2025-06-20

**Authors:** Silvia Selene Moreno-Guerrero, Arturo Ramírez-Pacheco, Luz María Rocha-Ramírez, Gabriela Hernández-Pliego, Alfonso Reyes-López, Juan José Luis Sienra-Monge, Luis Enrique Juárez-Villegas

**Affiliations:** 1 Departamento de Hemato-Oncología, Hospital Infantil de México Federico Gómez, Dr. Márquez No. 162, Col Doctores, Delegación Cuauhtémoc, Mexico City 06720, Mexico; sswitch@yahoo.com (S.S.M.-G.); artur_tauro@yahoo.com.mx (A.R.-P.); aleirbagy@hotmail.com (G.H.-P.); luisjuarezvillegas@gmail.com (L.E.J.-V.); 2 Unidad de Investigación en Enfermedades Infecciosas, Hospital Infantil de México Federico Gómez, Dr. Márquez No. 162, Col Doctores, Delegación Cuauhtémoc, Mexico City 06720, Mexico; 3 Centro de Estudios Económicos y Sociales en Salud, Hospital Infantil de México Federico Gómez, Dr. Márquez No. 162, Col Doctores, Delegación Cuauhtémoc, Mexico City 06720, Mexico; alfonso.reyes.lopez@outlook.com; 4 Subdirección de Pediatría Ambulatoria, Hospital Infantil de México Federico Gómez, Dr. Márquez No. 162, Col Doctores, Delegación Cuauhtémoc, Mexico City 06720, Mexico

**Keywords:** neuroblastoma, cytokines, serum levels, pro-inflammatory

## Abstract

**Background:** Cytokines are effector molecules of the host immune response that have been associated with chronic inflammatory processes related to risk and a poor prognosis in cancer patients. However, the impact of these molecules on the genesis and prognosis of Neuroblastoma (NB) is uncertain. **Objective:** The aim of the study was to analyze serum concentrations of pro-inflammatory and anti-inflammatory cytokines in a cohort of pediatric patients with NB. **Methods:** We evaluated the serum levels of several cytokines with a pro-inflammatory profile (IL-8, MCP-1, IL-6, IL-1β, IFN-γ, TNF-α, IL-12 p40 and IL-12p70), and anti-inflammatory profile (IL-10 and TGF-β), in pediatric patients with NB using the ELISA method, compared to a healthy control group (non-oncology). **Results:** Serum levels of pro-inflammatory cytokines IL-6, TNF-α, INF-γ, IL-12, IL-8 and MCP1 were significantly elevated in patients with NB compared to healthy pediatric controls. Only the anti-inflammatory cytokine IL-10 showed high levels in patients with NB in relation to the control group, unlike the synthesis of TGF-β, which had no differences between both groups. Likewise, significant positive correlations were found between the circulating levels of IL-6 with TNF-α (r = 0.667; *p* ≤ 0.01), IL-6 with IL-8 (r = 0.641; *p* ≤ 0.01), IL-8 with TNF-α (r = 0.637; *p* ≤ 0.01) and IL-10 with INF-γ (r = 0.542; *p* ≤ 0.01) in patients with NB. The simple logistic regression analysis revealed a significant association between low serum concentrations of IL-6 and a lower risk of presenting an unfavorable tumor histology (*p* = 0.048); in addition, low levels of IL-12p40 (*p* = 0.007), IFN-γ (*p* = 0.006) and MCP-1 (*p* = 0.029) were found to be associated with a lower risk of presenting NB in disseminated stages of the disease (INSS 3 and 4). Additionally, a higher risk of death was found in patients with high levels of IL-6 (*p* = 0.022) and IL-8 (*p* = 0.04). **Conclusions:** Taken together, the results demonstrate that the serum levels of pro-inflammatory cytokines such as IL-6, IL-8, IFN-γ, and TNF-α could be considered serum immunological indicators with a potential prognostic role in the pathogenesis of NB.

## 1. Introduction

Neuroblastoma (NB) is a tumor of early childhood and the most common malignancy diagnosed in the first year of life, with 25 to 50 cases per million individuals [1]. More than 90% of tumors occur in children under 10 years of age, with a mean age at diagnosis of 18 months [2,3]. NB accounts for 8–10% of all cancers in children aged 0–14 years in countries such as the United States of America, Australia and Europe; meanwhile, in Latin American countries and in some cities in Asia and India, the frequency is lower, at about 3%. In developed countries, the average annual incidence of NB in children varies between 7 and 14 per one million children/year; however, in developing countries, the incidence is low and has been reported to be less than 6 per one million children/year [4,5,6]. In low- and middle-income countries, most NB occurs in advanced stages. The survival of patients with low- and intermediate-risk disease is approximately 100%, and the 5-year survival rate of patients with high-risk NB is less than 50% [6]. There are some ethnic differences in NB, with the disease being more prevalent in those of European ancestry, and African-American children tending to have higher-risk disease [7]. NB in Mexico represents a frequency and incidence of 2.7 to 3.6% per one million children per year, with 80% of cases diagnosed in advanced stages of the disease [3,4]. The survival of patients who develop NB after the first year of life with metastasis does not show improvement compared to other neoplasms and continues to be a challenge for clinical professionals [3,5]. NB is a tumor derived from primordial neural crest cells that migrate during fetal development, which explains the multiple anatomical sites where this tumor can occur [2]. They are clinically heterogeneous tumors, with variability in location, histopathologic appearance, and biological characteristics. Their response to therapy and clinical evolution is variable, ranging from the spontaneous regression of the tumor to aggressive disease with metastatic dissemination [2,3,4,5]. The main factors influencing the clinical behavior of neuroblastomas are tumor stage, age at diagnosis, pathologic risk classification, cytogenetics, and molecular genetics [5,8]. These factors have been combined to define low-, intermediate-, and high-risk groups, which are used to define treatment strategies [2,5]. Various imaging studies such as radiographs, ultrasonography, computed tomography (CT) and magnetic resonance imaging (MRI) are used for the diagnosis and follow-up of this tumor, can find tumor calcifications and tumor masses affecting the anatomy, assess the extent of the primary tumor and study paravertebral tumors that present invasion of the spinal canal. In addition, gammagraphic studies can be used, since due to the secretory activity of NB, it captures precursors of pressor amines; in addition, isotope-labeled meta-iodobenzyl-guanidine (MIBG) helps tumor localization and eventually tumor treatment [2,5]. The staging of neuroblastoma tumors is performed based on clinical, radiographic, and surgical evaluation, according to the criteria of the International Neuroblastoma Staging System (INSS) and the International Neuroblastoma Risk Group (INRG) [5,8]. Histology and molecular analysis are very important factors in the evaluation of these patients. NB belongs to the group of small round blue cell tumors, is separated by fibrovascular septa with areas of necrosis and calcifications, and is associated with the presence of neuropil in its most immature form and in some cases the presence of Homer-Wright rosettes [5,8]. Different degrees of differentiation may be present, which is determined based on histopathologic criteria following the classification parameters of the International Neuroblastoma Pathology Classification (INCP) using histopathologic and immunohistochemical studies, such as detection of the NB84 marker, ploidy evaluation and molecular analysis [5,8]. NBs with poor stroma and highly undifferentiated cells have a worse prognosis than those rich in stroma and with differentiated cells. Histology according to the Shimada system is based on the amount of Schwannian stroma, the degree of differentiation, the mitosis–karyorrhexis index (MKI) and the age at diagnosis, classifying tumors into two groups with a favorable and unfavorable histologic prognosis [5,8]. Some other tools for the accurate diagnosis and staging of NB are the measurement of some biomarkers. The INSS recommends the determination of urinary catecholamine metabolites (vanillylmandelic acid and homovanillic acid) for diagnosis [9]. In addition, it considers the measurement of conventional serum markers such as neuron-specific enolase (NSE), ferritin and lactate dehydrogenase as an optional aid, although associated with limited diagnostic sensitivity and specificity [10]. However, authoritative clinical practice guidelines warn of the poor diagnostic performance of some of them, such as serum NSE determination. A recent study reported that there is no definitive evidence to support the use of serum NSE for the diagnosis and follow-up of NB [10]. The pathogenesis of NB is multifactorial and involves genetic alterations in the amplification of genes such as MYCN and ALK, polymorphisms in LIN28B and chromosomal alterations [5,8,11].

The immunological approach in the pathogenesis of NB is limited; however, the binomial between the activation of a persistent inflammatory response and cancer is hypothesized to be an important risk factor in the promotion and progression of tumors [12]. The inflammatory response involves the synthesis of different mediators, including cytokines; these proteins regulate cellular functions such as differentiation, cell migration and repair processes [13]. The inflammatory response is a physiological event of the immune system that is produced by a variety of infectious and non-infectious stimuli. These stimuli can activate acute and chronic inflammation in different organs of the host and cause tissue damage. In particular, the chronic inflammatory response identified by an uncontrolled cytokine storm can trigger cellular events associated with malignant transformation and carcinogenesis [14,15]. It is documented that cytokines produced by cellular interactions in the tumor microenvironment play an important role in cancer pathogenesis [14,15]. Non-malignant cells in the tumor microenvironment can positively or negatively affect the growth, survival and metastatic potential of tumor cells including NB [14,15,16]. Alternatively, malignant cells may respond to host-derived cytokines that promote growth, attenuate apoptosis, and facilitate invasion and metastasis [15]. Some experimental evidence points to the participation of inflammatory mediators such as the synthesis of TNF-α, IL-6, TGF-β and IL-10 in the initiation and progression of malignancy [15,16,17,18,19]. Likewise, the synthesis of inflammatory mediators such as pro-inflammatory cytokines and chemokines favors the secretion of other effector molecules such as hypoxia-inducible factor (HIF-1), vascular endothelial growth factors (VEGFs) and secondary mediators such as the activation of cyclooxygenase (COX) [12]. Consequently, the inflammatory response is regulated through the fine balance of pro-inflammatory and anti-inflammatory cytokines [12,20]. In this context, high concentrations of IL-6 are associated with a cellular microenvironment that benefits the growth of tumor cells in various types of cancer, such as lung, mammary gland, and colon, among others [21,22]. Previous studies have supported the hypothesis of anti-neuroblastoma immune responses supported by in vitro assays of the inhibition of NB cells with lymphocytes obtained from NB patients and the association of the expression profiles of anti-inflammatory cytokines such as IL-10 and TGFβ1, with M2 polarized macrophage combination. NB IL-6 and VEGF are the best-characterized cytokines that stimulate tumor growth and metastasis, while others such as IFN-γ may exert anti-NB activity by inducing tumor cell apoptosis and inhibiting angiogenesis. Likewise, transcriptional proteins such as NF-κB, and STAT3 are found to be dysregulated in several tumor types including neuroblastoma [14,23,24]. Several reports suggest that IL-6 and IL-10 play an important role in NB via the activation of recruited inflammatory cells, mainly by myeloid cells and fibroblasts that contribute to increased resistance to neuroblast cell growth, chemo-resistance, and immune evasion [23,24,25]. Other investigations demonstrate elevated levels of the soluble IL-6 receptor in correlation with the presence of metastasis in vivo experimental models of NB and elevated levels of IL-6 in mononuclear cells from high-risk NB patients [26]. In vitro and in vivo studies also demonstrate the effect of other cytokines, such as IFN-γ, by inhibiting the proliferation of NB cells by apoptosis or neuronal differentiation in NB cell lines [27]. Furthermore, under certain experimental conditions, cultures of NB cell lines with IFN-γ and TNF-α produce morphological and biochemical changes [27,28]. For these reasons, the balance of pro- and anti-inflammatory cytokines in host homeostasis and cancer prevention is potentially important.

Considering the diverse role of cytokines in the inflammatory response of cancer, this study aimed to analyze the profile of circulating pro-inflammatory and anti-inflammatory cytokines in the serum of a cohort of patients with NB. In this study, we evaluated the hypothesis that circulating levels of pro-inflammatory, chemokine, and anti-inflammatory cytokines could be use as early detection immunological biomarkers for a better understanding of the pathogenesis of NB.

## 2. Materials and Methods

### 2.1. Patients

This study included twenty-seven pediatric patients from the Federico Gómez Children’s Hospital of Mexico who were diagnosed with neuroblastoma from December 2016 to January 2020 based on histopathological findings in tumor biopsies, the use of immunohistochemical methods for differential diagnosis with other tumor types, the use of monoclonal antibodies including neuron-specific enolase (NSE), synaptophysin, ganglioside GD2, neural cell adhesion molecule (NCMAM), tyrosine kinase (TRK) and chromogranin A (CGA), morphological findings in bone marrow, and the evaluation of catecholamides in urine, according to the protocols established at the Institute. The staging of patients was performed based on clinical, radiographic, and surgical evaluation, according to the criteria of the International Neuroblastoma Staging System (INSS). A peripheral blood sample was obtained at the time of diagnosis, before starting chemotherapy or tumor resection. Clinical information such as the age at diagnosis, risk, stage and site of the primary tumor, histology, clinical follow-up time and prognostic factors of each patient were obtained from a review of clinical records. The normal pediatric controls were obtained with the prior informed consent of a parent or guardian of healthy children; a physical examination and review of their clinical history were performed, and infectious processes were identified in none of them, with no history of allergies or immune diseases at the time of sample collection.

### Ethical Considerations

This project was evaluated and approved by the Ethics Committee of the Hospital Infantil de México Federico Gómez, with number CONBIOÉTICA-09-CEI-010-20160627. The informed consent was signed by the legal guardians of all patients. This study was carried out in accordance with the principles established by the World Medical Assembly of Helsinki and all applicable modifications established by the World Medical Assemblies and the ICH guidelines for Good Clinical Practice (GCP), as well as the regulations of the General Law of Health in Research for the Health of Mexico.

### 2.2. Determination of MYCN Gene Amplification

For the detection of *MYCN* gene amplification, DNA was obtained from formalin-fixed, paraffin-embedded samples of NB using the QIAamp DNA FFPE Tissue kit (Qiagen, Hilden, Germany), according to the manufacturer’s instructions. The *MYCN* copy number was analyzed by real-time PCR, using the commercial TaqMan copy number assay for the *MYCN* gene (ID Hs00658058) from Applied Biosystems of Thermo Fisher Scientific (Thermo Fisher Scientific, Foster, CA, USA), in accordance with the manufacturer’s instructions.

### 2.3. Determination of Serum Levels of Pro-Inflammatory Cytokines, Chemokines and Anti-Inflammatory

Blood samples were obtained in tubes for serum analysis with separating gel, which was kept for 1 h for complete retraction of the clot and subsequently centrifuged for 15 min at 3500 rpm. The serum was stored at −70 °C in 500 µL aliquots for the subsequent analysis of immunological markers. Pro-inflammatory cytokines (IL-1β, TNF-α, IL-6, and IFN-γ), chemokines (IL-8, MCP-1) and anti-inflammatory cytokines (IL-10, TGF-β) were quantified in the serum of NB patients and controls by the ELISA method using the BD OptEIA ELISA Kit (BD Biosciences, Franklin Lakes, NJ, USA), according to the manufacturer’s instructions. The samples were analyzed in duplicate and the concentration was expressed in pg/mL and calculated by interpolation to a standard curve for each cytokine, considering the 1:5 dilution factor for each of them. To establish cut-off lines for each of them, we previously carried out a preliminary analysis that included 30 serum samples from the pediatric population and 3 negative controls to obtain the mean value of the determinations ± 2 standard deviations (SD).

### 2.4. Statistical Analysis

To compare the values of serum cytokine concentrations between the group of patients with NB and the control group, the distribution of these variables was first evaluated using the Shapiro–Wilk test to determine the use of parametric tests. If a normal distribution is found, non-parametric tests are used; however, because the distribution was not normal, the Wilcoxon test was used. Spearman’s p coefficient was used to evaluate the correlation between variables. Descriptive data were represented in terms of frequencies. Differences between the categorical variables of the clinical data of patients and cytokine levels were analyzed using Fisher’s exact test. To evaluate the association between serum cytokine levels and the presence of the disease and prognostic factors, we estimated the odds ratios (OR) using simple logistic regression analysis. Overall survival was calculated from the date of diagnosis to the date of death or last contact with the patient. It was performed using the Kaplan–Meier method and the differences between groups were estimated by the log rank test. The value of *p* < 0.05 was considered statistically significant. Statistical and graphic analyes were carried out using STATA version 13, SPSS version 19 and GraphPad Prism version 5 programs.

## 3. Results

### 3.1. Characteristics of the Patients

Twenty-seven patients diagnosed with neuroblastoma at the Department of Haemato-Oncology at our institute form December 2016 to January 2020 were included. Patient staging was performed based on clinical, radiographic, and surgical evaluation. The main clinical prognostic factors were evaluate; these were age at diagnosis, disease stage, histology and *MYCN*. Age was classified as <18 months (59.3%), 18 months–5 years (22.2%) and >5 years (18.5%). The diagnoses included 15 cases (55.6%) of high-risk neuroblastoma, 7 cases with an intermediate risk (25.9%) and 5 cases with a low risk (18.5%). The histology was classified as favorable and unfavorable according to the classification proposed by the International Neuroblastoma Pathology Committee (INPC) according to the Shimada method, with 12 (44.4%) presenting with unfavorable histology and 15 (55.6%) with favorable histology. In total, 55.5% presented metastasis at diagnosis, only 3.7% presented amplification of the *N-Myc* gene, 25.9% of patients presented relapse, and 22.2% died during follow-up. The control group included 27 healthy subjects and they were matched by age and sex with the group of patients with NB. The characteristics of the patients and the control group are described in Table 1.

### 3.2. Relationship of Serum Levels of Pro-Inflammatory, Anti-Inflammatory Cytokines and Chemokines Between NB Cases and Control Group

**Figure 1:**
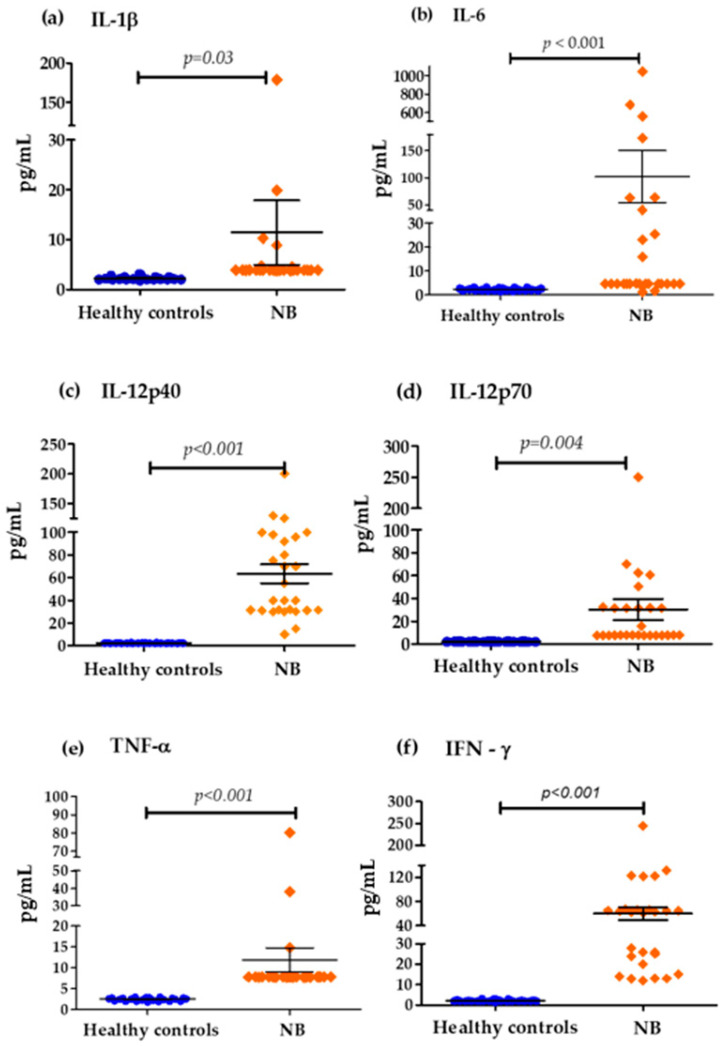
Serum concentrations of pro-inflammatory cytokines in the control group and patients with NB: (**a**) Differences in IL-1β concentrations (cut-off values; Low Levels ≤ 3.4 pg/mL—High Levels > 3.4 pg/mL), (**b**) Differences in IL-6 concentrations (cut-off values; Low Levels ≤ 7 pg/mL—High Levels > 7 pg/mL), (**c**) Differences in IL-12p40 concentrations (cut-off values; Low Levels ≤ 41 pg/mL—High Levels > 41 pg/mL), (**d**) Differences in IL-12p70 concentrations (cut-off values; Low Levels ≤ 65 pg/mL—High Levels > 65 pg/mL), (**e**) Differences in TNF-α concentrations (cut-off values; Low Levels ≤ 10 pg/mL—High Levels > 10 pg/mL), (**f**) Differences in IFN-ɤ concentrations (cut-off values; Low Levels ≤ 20 pg/mL—High Levels > 20 pg/mL).

The analysis of the levels of pro-inflammatory cytokines in the cohort of patients with NB, compared to the control group of healthy children, showed that the average concentration between both groups was as follows: IL-1β (11.438 pg/mL vs. 2.25 pg/mL), IL-6 (102.28 pg/mL vs. 2.32 pg/mL), IL-12p40 (63.48 pg/mL vs. 2.34 pg/mL), IL-12p70 (30.226 pg/mL vs. 2.196 pg/mL), TNF-α (11.851 pg/mL vs. 2.474 pg/mL) and IFN-γ (59.874 pg/mL vs. 2.185 pg/mL). It was observed that serum concentrations were significantly high in patients with NB compared to the healthy controls (Figure 1).

**Figure 2:**
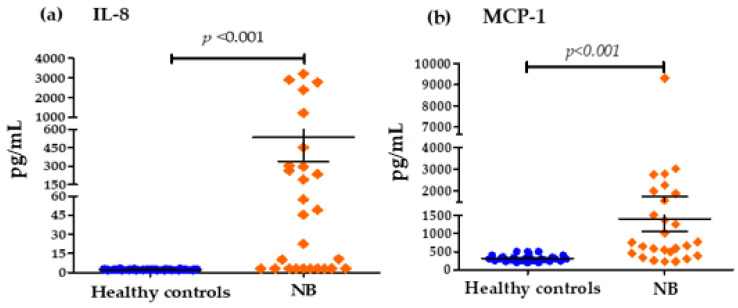
Serum concentrations of IL-8 and MCP1 chemokine groups: (**a**) Differences in IL-8 concentrations in pg/mL for the control group and patients with NB (cut-off values; Low Levels ≤ 40 pg/mL—High Levels > 40 pg/mL). (**b**) Differences in MCP1 concentrations in pg/mL for the control group and patients with NB (cut-off values; Low Levels ≤ 1000 pg/mL—High Levels > 1000 pg/mL).

**Figure 3:**
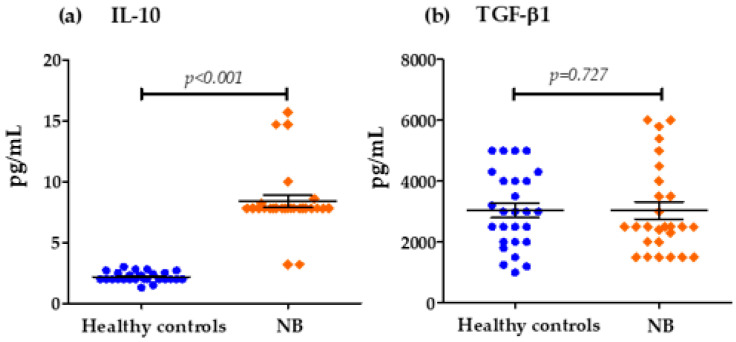
Serum concentrations of IL-10 and TGF-β chemokines groups: (**a**) Differences in IL-10 concentrations in pg/mL for the control group and patients with NB (cut-off values; Low Levels ≤ 8 pg/mL—High Levels > 8 pg/mL), (**b**) Differences in TGF-β concentrations in pg/mL for the control group and patients with NB (cut-off values; Low Levels ≤ 5000 pg/mL—High Levels > 5000 pg/mL).

Figure 2 shows that the concentrations of important chemokines such as IL-8 and MCP-1 were also elevated in NB patients at diagnosis with respect to the control group: IL-8 (535.63 pg/mL vs. 2.296 pg/mL) and MCP1 (1406.89 pg/mL vs. 314.62 pg/mL) (Figure 2). Figure 3 shows that the serum levels of anti-inflammatory cytokines in patients with NB at the time of diagnosis compared to healthy controls were only significantly elevated for IL-10 (8.389 pg/mL vs. 2.185 pg/mL). However, in the case of TGF-β, there were no important differences between both groups (3038.89 pg/mL vs. 3033.33 pg/mL).

### 3.3. Correlation of Serum Levels of Pro-Inflammatory, Anti-Inflammatory Cytokines and Chemokines in Patients with NB

We analyzed the potential for correlation between the serum levels of the various cytokines found in patients with NB at the time of diagnosis. Table 2 shows the significant positive correlation between the levels of IL-6 with TNF-α (r = 0.667; *p* ≤ 0.01), IL-6 with IL-8 (r = 0.641; *p* ≤ 0.01), IL-8 with TNF-α (r = 0.637; *p* ≤ 0.01) and IL-10 with INF-γ (r = 0.542; *p* ≤ 0.01). These data show that IL-8 levels present a statistically significant and proportional linear association with the serum levels of IL-6 and TNF-α in patients with NB. TNF-α also showed a positive correlation with IL-6 levels. Furthermore, the concentrations of IL-12p40 and IL-12p70 also had a significant correlation (r = 0.742, *p* ≤ 0.01), indicating that both vary in the same direction. The other cytokines analyzed did not present any significant correlation between them. Although IL-10 is one of the major anti-inflammatory cytokines, which exerts its action through the inhibition of IL-1, IL-6 and TNF-α synthesis in this study, the correlation coefficients for IL-10 with IL-1, IL-6 and TNF-α (−0.08, −0.112, −0.061) were negative; however, values close to 0 indicate that there is no linear correlation, and in the analysis, these correlations were not statistically significant (Table 2).

### 3.4. Analysis of Serum Levels of Pro-Inflammatory and Anti-Inflammatory Cytokines and Chemokines and Prognostic Factors of Patients with NB

For the association and simple logistic regression analyses, the circulating levels of each cytokine were stratified into low and high levels, according to the cut-off values established for each of them (Table A1). The analysis of clinicopathological factors with the serum levels of pro-inflammatory cytokines evaluated in this study showed a significant association (*p* = 0.022) between IL-6 levels and tumor differentiation; this is observed in Table A2, where 44.4% of patients with low serum levels of this cytokine presented partially differentiated neuroblastic tumors. In addition, most patients with a favorable tumor histology presented low levels of IL-6 (44.4%) and TNF-α (55.6%); however, these associations were not significant *p* = 0.05 and *p* = 0.07 respectively (Table A2). The other pro-inflammatory cytokines analyzed showed no association with the clinical and pathological characteristics of patients with NB (Table A2). The analysis of the chemokines evaluated only showed a relationship with tumor differentiation; in the case of MCP-1, 40.7% of patients with low levels presented partially differentiated tumors with a significant association (*p* = 0.027); similarly, 33.3% of patients with partially differentiated tumors had low levels of IL-8. However, this association was not significant (*p* = 0.09) (Table A3). In relation to the analyzed anti-inflammatory cytokines IL-10 and TGF-β, the serum levels of these did not present a significant relationship with the clinical data of the patients (Table A4).

### 3.5. Association of Serum Cytokine Levels with the Clinicopathological Characteristics of Patients with NB

A simple logistic regression analysis was performed to evaluate the association between the serum levels of the anti-inflammatory and pro-inflammatory cytokines and chemokines evaluated in patients with NB and the risk of presenting prognostic factors, relapse, and death. This analysis showed that low levels of IL-6 were significantly associated with a lower risk of presenting an unfavorable tumor histology (*p* = 0.048; OR = 0.17, 95% CI = 0.032–0.98) and that high levels were associated with a higher risk of death (*p* = 0.022; OR = 15.99, 95% CI = 1.49–171.2) (Table 3). Furthermore, low serum levels of IL-12p40 (*p* = 0.007; OR = 0.08, 95% CI = 0.01–0.5) and IFNγ (*p* = 0.006; OR = 0.18, 95% CI = 0.03–1.07) were associated with a decrease in the risk of presenting neuroblastoma in disseminated INSS stages 3 and 4 (Table 3). The other concentrations of the pro-inflammatory cytokines evaluated did not present significant associations with any clinicopathological factor.

A simple logistic regression analysis for serum chemokine concentrations showed that low serum levels of MCP-1 (*p* = 0.029; OR = 0.15, 95% CI = 0.02–0.82) and IL-8 (*p* = 0.05; OR = 0.20, CI 95% = 0.03–1.05) were associated with a decrease in the risk of presenting neuroblastoma in INSS stage 3 and 4; however, for IL-8, the *p* value remained at the limit required to be considered statistically significant (Table 4). On the other hand, high levels of IL-8 were associated with a higher risk of death (*p* = 0.04; OR = 6.66, 95% CI = 0.65–67.47) (Table 4).

In the case of anti-inflammatory cytokines, the simple logistic regression analysis showed significant associations with the INSS stage. It was observed that low serum concentrations of IL-10 (*p* = 0.013; OR = 0.09, 95% CI = 0.01–0.6) and TGF-β (*p* = 0.04; OR = 0.11, 95% CI = 0.01–0.97) were associated with a decrease in the risk of presenting neuroblastic tumors in localized INSS stages 1 and 2 (Table 5). The other variables did not present associations with the serum levels of the cytokines evaluated. In the case of anti-inflammatory cytokines, the simple logistic regression analysis showed significant associations with the INSS stage. It was observed that low serum concentrations of IL-10 (*p* = 0.013; OR = 0.09, 95% CI = 0.01–0.6) and TGF-β (*p* = 0.04; OR = 0.11, 95% CI = 0.01–0.97) were associated with a decrease in the risk of presenting neuroblastic tumors in localized INSS stages 1 and 2 (Table 5). The other variables did not present associations with the serum levels of the cytokines evaluated.

### 3.6. Survival Analysis

**Figure 4:**
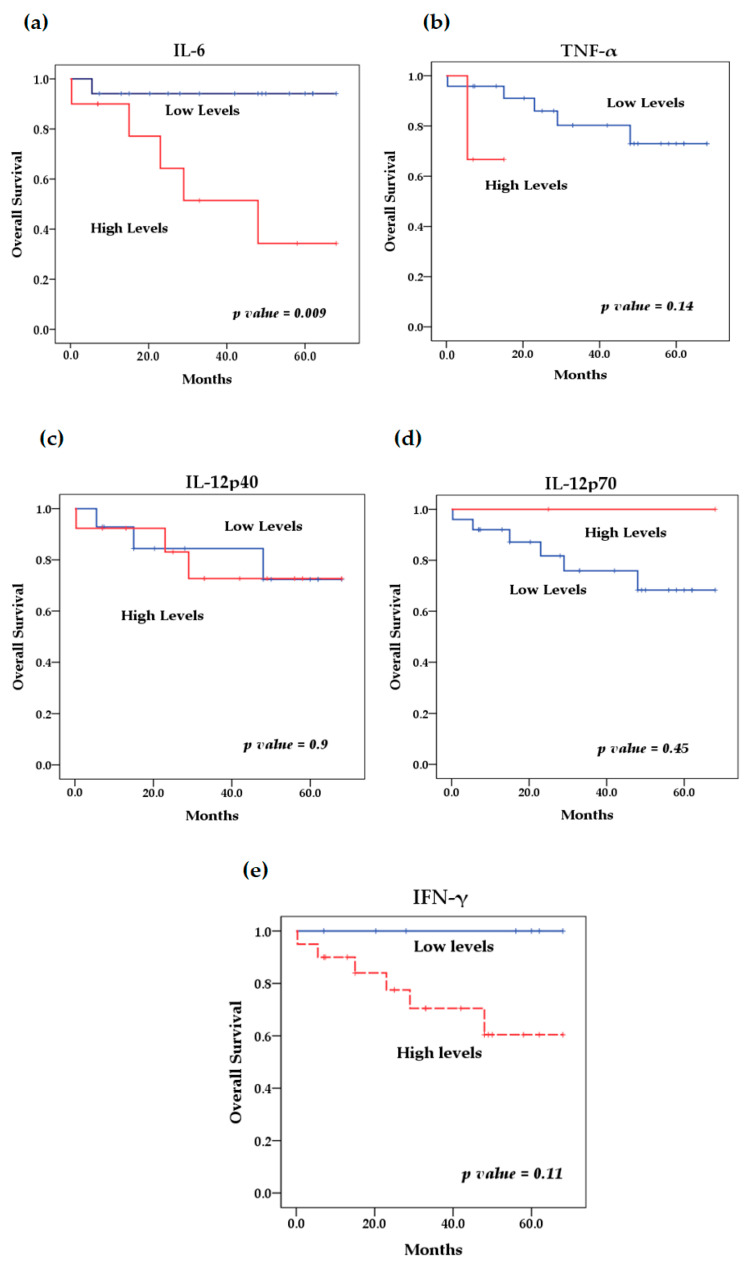
Kaplan–Meier curves comparing patients with high levels to those with low levels of pro-inflammatory cytokines: (**a**) IL-6, (**b**) TNF-α, (**c**) IL-12p40, (**d**) IL-12p70, (**e**) IFN-γ.

**Figure 5:**
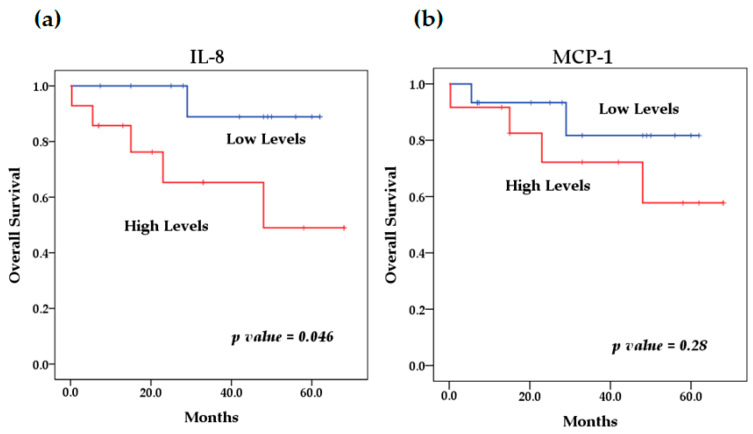
Kaplan–Meier curves comparing patients with high levels to those with low levels of chemokines: (**a**) IL-8, (**b**) MCP-1.

**Figure 6:**
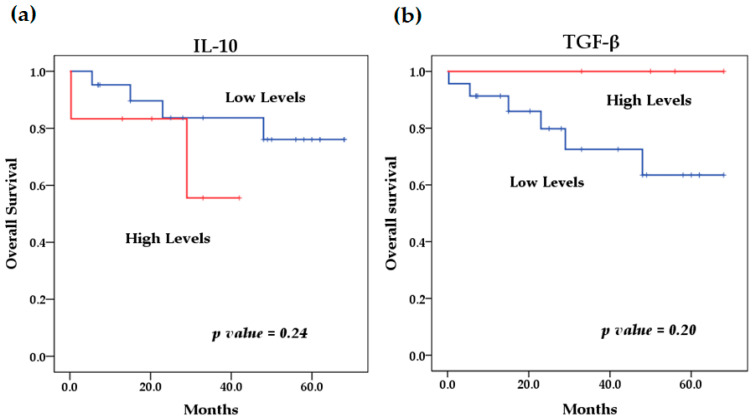
Kaplan–Meier curves comparing patients with high levels to those with low levels of anti-inflammatory cytokines: (**a**) IL-10, (**b**) TGF-β.

We evaluated the levels of cytokines at diagnosis and their relationship with overall survival. The survival analysis using the Kaplan–Meier method showed that patients with high levels of IL-6 (*p* = 0.046) (Figure 4) and IL-8 (*p* = 0.009) (Figure 5) at diagnosis presented a significantly lower overall survival rate compared to patients with low levels of these cytokines; these results were previously reported by our group [29]. The rest of the serum levels of pro-inflammatory, anti-inflammatory cytokines and chemokines analyzed did not show a significant association with overall survival in patients with NB (Figure 4, Figure 5 and Figure 6).

## 4. Discussion

The relationship between inflammation and cancer has been recognized since the seventeenth century [30]. There is clear evidence that inflammatory growth factors play a vital role in the development and progression of various malignant neoplasms [12,30,31,32]. Cytokines are an important component of these factors, and have been associated with certain types of cancer [15,16]. In addition, solid tumors can initiate and perpetuate local inflammatory processes to promote tumor growth and dissemination; through several mechanisms, many of them, via inflammatory mediators such as cytokines, which are important growth and survival factors, stimulate premalignant cell survival and proliferation. In addition, inflammatory mediators often activate oncogenic transcription factors such as NF-κB and STAT3, while inflammation can stimulate tumor angiogenesis, tumor invasiveness and metastatic dissemination. Moreover, tumor-associated inflammation can suppress the antitumor immune response and divert tumor-specific immune cells from being anti-tumorigenic to becoming pro-tumorigenic [33,34,35]. In the amplification of the immune response mediated by the release of cytokines, early homeostasis must be achieved in order to not cause damage to the host due to exacerbated secretion [35].

In the genesis of NB, clinical studies investigating cytokine levels are rare. In our study we showed that pro-inflammatory cytokines such as IL-8, IL-6, IL-12, TNF-α and IFN-γ, and the chemokines IL-8 and MCP-1, were significantly higher in NB patients compared to healthy controls (Figure 1 and Figure 2). The elevated expression of several cytokines such as IL-1β, IL-6, IL-10, TNF-α, and TGF-β has been reported to be involved in tumor initiation and progression in various types of cancer [36]. Likewise, high concentrations prior to the diagnosis of inflammation markers such as C-reactive protein have been reported, and several cytokines such as IL-1, IL-6, IL-8, TNF-α, among others, have been associated with a high risk of developing some types of cancer including breast, lung, prostate, ovarian and colorectal cancer [37,38].

In most tumors, IL-10 and TGF-β are identified as immunosuppressive cytokines that increase tumor growth. IL-10 and TGFβ are anti-inflammatory cytokines that, on the one hand, control the severity of autoimmune and inflammatory reactions and, on the other hand, play a negative role by damaging the host’s anti-tumor effector immunity and creating a favorable environment for tumor growth and metastasis [16]. It has been reported that the tumor microenvironment is mainly dominated by immunosuppressive cytokines that control anti-tumor effector immunity and promote the survival and proliferation of cancer cells, leading to increased tumor growth. In addition to tumor cells, the heterogeneous immune cells present in the tumor environment are an important source of immunosuppressive cytokines [16]. IL-10 is an anti-inflammatory cytokine secreted by various immune cells that regulate excessive inflammatory responses by promoting tissue repair mechanisms and preventing autoimmune diseases [39]. In solid tumors, the involvement of IL-10 is complex in its interaction with tumor cells, and it has been suggested that elevated serum levels of IL-10 and other markers in patients with brain glioma could be a valuable prognostic indicator [40]. In our study, the anti-inflammatory cytokine IL-10 showed high serum concentrations in patients with NB compared to the control group (Figure 3); this agrees with other authors who found a high expression of IL-10 mRNA in patients with metastatic NB [24]. Likewise, these authors suggested that the plasma levels of IL-10 were high in patients with NB regardless of the stage of the disease. Also, in other studies, it has been suggested that IL-10 and TGF-β exert an important role in the invasion and migration of NB [41]. In addition, other authors, based on in vitro models, have analyzed the participation of cytokines in the tumor microenvironment in NB samples in relation to components of the immune response, including macrophages, noting that stage I tumors in NB patients have a high expression of markers associated with M2-type macrophages (IL-4, IL-10, and TGF-β), unlike stage IV tumors, with the expression of IL1-β and TNF-α cytokines associated with poor prognosis [42]. In contrast, other previous reports in patients with prostate cancer support the idea that circulating levels of IL-10 do not contribute to the relative risk of this type of cancer [43]. TGF-β plays a dual role; it inhibits cell proliferation and promotes apoptosis, which helps control inflammation, but on the other hand, it also promotes tumor growth, invasion, and metastasis [39,40,41,44,45]. Previous studies have indicated that TGF-β cytokine signaling can enhance cell proliferation and metastatic spread in the tumor microenvironment and inhibit immune surveillance [46]. In prostate cancer patients, this cytokine promotes tumorigenesis by modulating the tumor microenvironment to epithelial–mesenchymal transition, making it an important factor in this type of cancer [45,47]. In addition, the pleiotropic characteristics of TGF-β contribute to drug resistance and weaken the response to treatment [48,49]. Furthermore, increased plasma levels of TGF-β in various types of cancer, such as multiple myeloma, at the time of diagnosis could be a predictive and favorable factor for the immune response in the host [50,51]. However, in patients with hepatocellular carcinoma, low levels of circulating TGF-β increase the risk of death [52]. In contrast, in this study, we found no significant differences in the levels of the anti-inflammatory cytokine TGF-β between NB patients and healthy controls, even though the expression of this cytokine has also been correlated with tumorigenesis, metastasis, and tumor recurrence. Furthermore, TGF-β has been shown to alter the lineage plasticity of CD8+ T cells in the tumor environment and make them more susceptible to TGF-β-induced immunosuppression [53]. Several studies have shown that the immunosuppressive tumor environment is primarily responsible for tumor growth and tumor resistance to multiple chemotherapeutic and immunotherapeutic drugs. It has been reported that acquired resistance to conventional immunotherapeutic treatment, such as immune checkpoint blockade, is predominantly due to increased immunosuppressive cytokines in the tumor environment [54,55]. These immunosuppressive cytokines restrict the proliferation and effector functions of antitumor immune cells, alter their plasticity, and convert them into suppressor phenotypes. Due to restricted infiltration or the absence of effector immune cells, immune checkpoint blockades cannot exert optimal effects [56,57,58]. On the other hand, we also found significant associations between low serum levels of IL-10 and TGF-β and a decreased risk of developing INSS stage 1 and 2 neuroblastoma tumors (Table 5). Therefore, it is crucial to study the role and effect of anti-inflammatory cytokines such as IL-10 and TGF-β in various tumors such as Neuroblastoma, because the development of immunotherapeutic drugs to combat immunosuppression within the tumor environment is currently of great importance. Blocking immunosuppressive cytokines such as IL-10 and TGF-β could play an essential role in sensitizing tumors to conventional therapies. Therefore, targeting highly abundant immunosuppressive cytokines such as IL-10 and TGF-β in the tumor microenvironment significantly helps to reverse immunosuppression, improving the efficacy of conventional cancer therapies, activating antitumor immunity, and controlling tumorigenesis.

In the context of pro-inflammatory cytokines, high levels of IL-8 and its association with neoplastic diseases have been demonstrated for several types of cancer [59]. IL-8 is a member of the family of chemokines and is known for its chemotactic properties in the initiation and amplification of the inflammatory reaction, as well as its role in the chronic inflammatory process as a pro-inflammatory cytokine [60,61]. Supporting our data, previous studies have reported a high expression of IL-8 and its receptors in NB biopsy samples [62]. The same authors of this research point out that the combination of IL-8 and its receptor, together with the in vitro expression of these molecules in NB cell lines, could be involved in the in vivo regulation of IL-8 in human NB. Furthermore, the role of IL-8 in paracrine stimulation promoting angiogenesis in several groups of solid tumors has been demonstrated in previous reports [63]. Interestingly, in our study, we observed that most patients with high levels of IL-8 presented tumors in disseminated stages (INSS3 and INSS4) (Table 4). Likewise, elevated levels of this chemokine were associated with an increased risk of death. This finding is consistent with research that indicates that IL-8 is produced mainly by cancer cells. It has been shown that serum levels of IL-8 correlate with tumor progression and act as a negative prognostic factor in several types of cancer [29,60,64]. We also found significantly higher levels of chemokine MCP-1 in NB patients. MCP-1 is a chemokine identified for the first time in human gliomas and myelo-monocytic cells, being a protein that regulates the recruitment of monocytes–macrophages and other cells at sites of inflammation [65]. Different studies have shown their critical role in acute inflammatory responses, which are particularly important in the development of cancer [66]. In models of NB cell lines, such as the cell line SN-N-AS, an increase in MCP-1 mRNA expression was observed when the cells were stimulated with sphingosine-1, suggesting part of the signaling pathways in the NB [67]. Additionally, we found that low circulating levels decrease the risk of presenting disseminated stages of the tumor (Table 4), which is why, in accordance with previous studies, the increased production of MCP1 is probably necessary for tumor development.

Interleukin 6 is another cytokine that we find elevated in the serum of NB patients; this cytokine has dual functions when also considering a cytokine with anti-inflammatory properties [68]. IL-6 facilitates the progression of some types of cancer via the involvement of certain biological mechanisms and cellular processes that involve apoptosis, survival, angiogenesis, invasiveness, metastasis, and metabolism [23,68,69,70]. Some reports have identified high levels of IL-6 as a marker of poor prognosis in some malignancies including NB [26,71,72,73]. In our investigation, the mean serum IL-6 concentrations were significantly higher in the NB patients than in the non-cancer control group (Figure 1). In addition, among the patients who presented high levels, the majority had high-risk NB, and low levels of this cytokine were associated with a decrease in the risk of presenting an unfavorable tumor histology (Table 3), which would support the prognostic value of this cytokine. In addition, high levels of this cytokine were also associated with an increased risk of death (Table 3). We also show a strong positive correlation between the serum levels of IL-6 with IL-8 and IL-6 with TNF-α in our cohort of NB patients (Table 2).

We found significant differences in the serum levels of IL-1β, IL-6, IL-12p40, IL-12p70, TNF-α, IFN-γ, IL-8 and MCP1 between patients with neuroblastoma and the control group. Other investigations have described differences in the serum cytokine profile in some diseases such as lung cancer, colorectal cancer and osteosarcoma, among others [64,74,75]. Elevated circulating concentrations of pro-inflammatory cytokines and chemokines in the serum of NB patients compared to controls in this study could be indicative of an inflammatory microenvironment, although their role in promoting NB is still controversial. A strong correlation was presented between the production of IL-6 with IL-8 and TNF-α; the relationship between these cytokines has been reported in other diseases such as breast cancer, where the levels of IL-6, IL-8 and TNF-α were correlated with the clinical disease stage and lymph node metastasis [76]. Likewise, it has been reported that TNF-α can behave as a serum indicator of gastric inflammation and malignant transformation [77]. Other authors indicated that IL-6, IL-8 and TNF-α are associated with an increased risk of osteosarcoma, and that elevated levels of IL-8 and TNF-α are correlated with the progression of this disease [75]. On the other hand, we also found a moderate correlation between IFN-γ and IL-10 (Table 2). This relationship has been reported in some studies on colon carcinoma, where they suggest that in certain colon carcinoma cells, the production of IL-10 derived from the tumor is directly regulated by the systemic or local production of pro-inflammatory cytokines, such as IL-6 and IFN-γ [78].

Furthermore, several studies have suggested that the increase in serum levels of various pro-inflammatory cytokines in cancer patients is associated with adverse disease outcomes [29,79,80]. We performed an analysis of the association between clinicopathological prognostic factors and the levels of cytokines, stratifying the serum concentrations of these into low levels and high levels. Considering the cut-off values previously established for each of them, we observed significant associations between low levels of IL-6 and a lower risk of presenting an unfavorable tumor histology and high levels of IL-6 and a higher risk of death. On the other hand, we found significant associations between low serum concentrations of IL-12p40 and MCP-1 and a decrease in the risk of presenting neuroblastoma in advanced stages INSS 3 and 4 (Table 3 and Table 4). In the case of anti-inflammatory cytokines, we found significant associations between low serum levels of IL-10 and TGF-β and a decrease in the risk of presenting neuroblastic tumors in stage INSS 1 and 2 (Table 5). These associations related to the histology of the tumor and the stages based on the INSS staging system are important because the treatment of NB must be carried out based on risk groups that are defined by biochemical, genetic and clinical prognostic factors, which are associated with patient survival. Among the most important clinical factors are the patient’s age, the location and histology of the tumor, and the stage based on the INSS criteria.

On the other hand, the high expression of several of these cytokines has been reported to predict poor survival in cancer patients. In this study, survival analysis showed that patients with elevated levels of several cytokines such as IL-6, IFN-γ, TNF-α, IL-8, MCP-1 and IL-10 had lower overall survival compared to patients with low serum levels. However, the result was only significant for elevated circulating levels of IL-6 (*p* = 0.046) and IL-8 (*p* = 0.009), which we previously reported; the identification of elevated circulating levels of IL-6 and IL-8 was associated with poor overall survival in NB patients [81]. Although it has been observed that patients with lower overall survival have elevated levels of some of these cytokines, as in the case of MCP-1, IL-10 and TNF-α, with the latter recently being reported by our group [82], this relationship was not significant for any of these cytokines, probably because the cut-off values used to stratify the serum concentrations of each of them into low and high levels were previously established in a pediatric population between 0 and 17 years of age and because both the control group and patients with NB were in an age range of 0 to 8 years; for this reason, we suggest that the age range could affect the stratification, because the expression profile of several of these cytokines may show an increase associated with age, which has been previously reported by other authors [74,75]. Therefore, the findings of this preliminary study can be taken as a basis for future studies with larger sample sizes, considering the age group of patients and cut-off values for the analysis of the expression of various cytokines and their possible association with survival. However, this does not underestimate the rest of the findings of this investigation, such as the significant differences found in the expression levels of the cytokines analyzed between patients with NB and healthy controls; this is because these groups were matched for age and sex to eliminate possible biases in the expression of these cytokines due to these factors.

In this study, we measured the serum concentrations of anti-inflammatory and pro-inflammatory cytokines and chemokines at the time of diagnosis. Therefore, we suggest that it would be important to evaluate the serum levels of cytokines in larger cohorts, at various times in the evolution of the disease (at diagnosis, after chemotherapy or tumor resection, at relapse, etc.), to examine whether an increase in the circulation of these cytokines can affect the development, response to treatment and prognosis of NB. It is also necessary to consider previous research indicating that age clearly influences the cytokine expression profiles of healthy children, reporting that several cytokines show an increase associated with age [83,84]. Likewise, the effect of gender on the serum and plasma concentrations of many cytokines has also been reported [84,85]. Therefore, in our study, the control group of healthy children was matched for age and gender with the group of patients with NB. In addition, serum cytokine levels may vary by race and contribute to cancer development differently among different ethnic groups [86]. This suggests that childhood cytokine expression dynamics and racial groups should be considered when evaluating them in diagnostic assays or as immunological biomarkers [83,84].

Cytokines regulate several steps in tumorigenesis, and some are also involved in resistance to chemotherapy. For this reason, various investigations have focused on the search for biochemical products related to tumors; in this case, the evaluation of circulating levels of various cytokines and the feasibility of being able to detect them in circulation in a blood test is very convenient. Therefore, determining the serum cytokine level profile combined with genetic markers and already established clinical data would allow the detection of NB in the early stages of its development, it diagnosis, and its appropriate treatment, in accordance with the tumor risk stratification; this would lead to significant reductions in morbidity and mortality related to NB.

Several authors have described the cellular and molecular functions of inflammation in cancer development, as well as the role of both tumor cells and other types of cells in the tumor microenvironment that participate in the production and release of growth factors and cytokines that modulate the inflammatory environment in tumor tissues [38,81,87,88]. Our data suggest that the microenvironment at the systemic level is mainly pro-inflammatory, and that these levels could be considered as strong candidates for immunological biomarkers useful in the prognosis of the pathogenesis of human neuroblastoma. However, additional investigations on a larger scale would be necessary to validate these findings and evaluate the origin of this cytokine dysregulation of pro-inflammatory cytokines (IL-8, IL-6, IFN-γ, and TNF-α) and anti-inflammatory cytokines (IL-10, TGF-β), providing further insight into the immune mechanisms involved in regulating the pro-inflammatory and immunosuppressive cell phenotype in NB; it is also necessary to perform studies to monitor their systemic effect versus localized effect and verify whether these cytokines can induce any effect on the response to treatment in patients with NB.

## 5. Conclusions

The interaction between malignant cells and cells of the tumor microenvironment can be dependent on contact, dependent on the host, or both. Soluble mediators of these interactions involve cytokine patterns.

We identified a particular pro-inflammatory profile in patients with NB, finding significantly elevated serum levels of IL-8, IL-6, IL-12, TNF-α and IFN-γ compared to healthy controls; this suggests their involvement in the development of this tumor and the possibility that a differentially expressed serum cytokine profile may be useful to detect the presence of NB, allowing early diagnoses. Correlating them with clinical stage may help guide the prognosis and treatment of patients. In addition, we found a strong correlation between IL-6 production and IL-8 and TNF-α. Elevated circulating levels of IL-6 were associated with an unfavorable tumor histology, and low serum levels of both IL-6 and IL-8 were associated with a lower risk of death.

Our results suggest that the serum levels of pro-inflammatory cytokines IL-6, IL-8, IFN-γ and TNF-α could be an immunological biomarker contributing to our understanding of the pathogenesis of neuroblastoma with prognostic potential in Mexican pediatric patients with NB. This is useful for identifying high-risk patients and monitoring the disease. Because these results lay the groundwork for the promotion of the clinical value of cytokine profiles, larger studies are needed in the future to examine their potential use as markers of changes in serum cytokine levels according to progression, tumor prognosis and response to treatment in NB. This would help stratify patients who are likely to progress rapidly and identify patients with poor prognosis, to provide them with intensive treatments that favor the success of the therapy and consider the use of immunotherapy.

## Figures and Tables

**Table 1:** Characteristics of patients with neuroblastoma and healthy subjects.

Characteristics		Patients n = 27 (%)	Healthy Controls n = 27 (%)
Age	<18 months	16 (59.3)	16 (59.3)
	18 months–5 years	6 (22.2)	6 (22.2)
	>5 years	5 (18.5)	5 (18.4)
Sex	Male	10 (37.03)	10 (37.03)
	Female	17 (62.97)	17 (62.97)
INSS stage	1	5 (18.5)	-----
	2b	2 (7.5)	-----
	3	7 (25.9)	-----
	4	13 (48.1)	-----
	4S	0 (0)	-----
INRG	L1	5 (18.5)	-----
	L2	8 (29.6)	-----
	M	14 (51.9)	-----
	MS	0 (0)	-----
Risk	Low	5 (18.5)	-----
	Intermediate	7 (25.9)	-----
	High	15 (55.6)	-----
Primary Tumor Site	Adrenal	8 (29.6)	-----
	Retroperitoneal	11 (40.7)	-----
	Paraspinal	3 (11.1)	-----
	Abdomen/Pelvic	1 (3.7)	-----
	Mediastinal	4 (14.9)	
Differentiation	Undifferentiated	2 (7.4)	-----
	Partially differentiated	16 (59.3)	-----
	Differentiated	5 (18.5)	-----
	Not specified	4 (14.8)	-----
Histology (Shimada)	Favorable	15 (55.6)	-----
	Unfavorable	12 (44.4)	-----
MYCN	Amplified	1 (3.7)	-----
	Not amplified	26 (96.3)	-----
Metastasis	Yes	15 (55.5)	-----
	No	12 (44.5)	-----
Relapse	Yes	7 (25.9)	
Death	Yes	6 (22.2)	

INSS = International Neuroblastoma Staging System, INRG = International Neuroblastoma Risk Group, MYCN = MYCN proto-oncogene.

**Table 2:** Correlation analysis of serum levels of pro-inflammatory and anti-inflammatory cytokines and chemokines in neuroblastoma patients.

Cytokines (pg/mL)	IL-1β	IL-6	IL-12 p40	IL-12 p70	TNFα	IFNγ	IL-8	MCP1	IL-10
IL-1β	----								
IL-6	−0.055	----							
IL-12 p40	−0.162	−0.068	----						
IL-12 p70	−0.105	−0.092	0.742 *	----					
TNFα	0.071	0.667 *	−0.191	−0.132	----				
IFNγ	−0.002	−0.196	0.164	−0.028	−0.162	----			
IL-8	−0.036	0.641 *	−0.227	−0.215	0.637 *	−0.281	----		
MCP1	0.000	0.359	−0.091	−0.073	−0.138	0.047	0.287	----	
IL-10	−0.080	-0.112	0.010	−0.046	−0.061	0.542 *	−0.268	0.034	----
TGF-β1	0.292	0.116	−0.096	−0.180	−0.198	−0.099	0.256	0.279	−0.358

* Significance level ≤ 0.01.

**Table 3:** The association between the serum levels of pro-inflammatory cytokines stratified by low levels versus high levels and the clinicopathologic factors of NB patients.

	IL-6		TNF-α		IL-12p40		IL-12p70		IFN-γ	
Variables	Low Levels	High Levels	Low Levels	High Levels	Low Levels	High Levels	Low Levels	High Levels	Low Levels	High Levels
Risk										
Low/Intermediate	10 (37)	2 (7.4)	10 (37)	2 (7.4)	5 (18.5)	7 (25.9)	11 (40.7)	1 (3.7)	2 (7.4)	10 (37)
High	7 (25.9)	8 (29.6)	14 (51.9)	1 (3.7)	9 (33.3)	6(22.2)	14 (51.9)	1 (3.7)	5 (18.5)	10 (37)
*p* Value	0.105		0.33		0.18		0.78		0.12	
OR (95% CI)	4.5 (0.73–27.7)		0.3 (0.023–3.79)		0.34 (0.06–1.68)		0.66 (0.03–11.93)		0.16 (0.01–1.64)	
INSS stage										
stage 1/2	5 (18.5)	2 (7.4)	7 (25.9)	0	2 (7.4)	5 (18.5)	7 (25.9)	0	2 (7.4)	5 (18.5)
stage 3/4	12 (44.4)	8 (29.6)	17 (63)	3 (11.1)	8 (29.6)	12 (44.4)	18 (66.6)	2 (7.4)	5 (18.5)	15 (55.6)
*p* Value	0.46		NC		0.007		NC		0.006	
OR (95% CI)	0.51 (0.08–3.12)		NC		0.08 (0.01–0.5)		NC		0.18 (0.03–1.07)	
Histology										
Favorable	12 (44.4)	3 (11.1)	15 (55.6)	0	7 (25.9)	8 (29.6)	13 (48.1)	2 (7.4)	5 (18.5)	10 (37)
Unfavorable	5 (18.5)	7 (25.9)	9 (33.3)	3 (11.1)	7 (25.9)	5 (18.5)	12 (44.4)	0	2 (7.4)	10 (37)
*p* Value	0.048		NC		0.54		NC		0.33	
OR (95% CI)	0.17 (0.032–0.98)		NC		1.6 (0.34–7.4)		NC		0.4 (0.06–2.56)	
MYCN										
Amplified	0	1 (3.7)	1 (3.7)	0	1 (3.7)	0	1 (3.7)	0	0	1 (3.7)
Not amplified	17 (63)	9 (33.3)	23 (85.2)	3 (11.1)	13 (48.1)	13 (48.1)	24 (88.9)	2 (7.4)	7 (25.9)	19 (70.4)
*p* Value	NC		NC		NC		NC		NC	
OR (95% CI)	NC		NC		NC		NC		NC	
Relapse										
Yes	3 (11.19	4(14.8)	7 (25.9)	0	4 (14.8)	3 (11.1)	6 (22.2)	1 (3.7)	2 (7.4)	5 (18.5)
No	14 (51.9)	6(22.2)	17 (63)	3 (11.1)	10 (37)	10 (37)	19 (70.4)	1 (3.7)	5 (18.5)	15 (55.6)
*p* Value	0.21		NC		0.74		0.43		0.85	
OR (95% CI)	3.11 (0.52–18.38)		NC		1.33 (0.23–7.55)		0.31 (0.01–5.85)		1.2 (0.17–8.24)	
Death										
Yes	1 (3.7)	5 (18.5)	5 (18.5)	1 (3.7)	3 (11.1)	3 (11.1)	6 (22.2)	0	0	6 (22.2)
No	16 (59.3)	5 (18.5)	19 (70.4)	2 (7.4)	11 (40.7)	10 (37)	19 (70.4)	2 (7.4)	7 (25.9)	14 (51.9)
*p* Value	0.022		0.62		0.91		NC		NC	
OR (95% CI)	15.99 (1.49–171.2)		1.9 (0.14–25.44)		1.1 (0.17–6.75)		NC		NC	

OR = unadjusted odds ratio; CI = confidence interval; NC = not calculable.

**Table 4:** The association between serum levels of chemokines stratified by low levels versus high levels and the clinicopathologic factors of NB patients.

	IL-8		MCP-1	
Variables	Low Levels	High Levels	Low Levels	High Levels
Risk				
Low/Intermediate	5 (18.5)	7 (25.9)	5 (18.5)	7 (25.9)
High	8 (29.6)	7 (25.9)	10 (37)	5 (18.5)
*p* Value	0.81			0.10
OR (95% CI)	0.83 (0.17–3.88)		0.25 (0.05–1.31)	
INSS stage				
stage 1/2	10 (37)	4 (14.8)	12 (44.4)	4 (14.8)
stage 3/4	3 (11.1)	10 (37)	3 (11.1)	8 (29.6)
*p* Value	0.05		0.029	
OR (95% CI)	0.20 (0.03–1.05)		0.15 (0.02–0.82)	
Histology (Shimada)				
Favorable	8 (29.6)	7 (25.9)	8 (29.6)	7 (25.9)
Unfavorable	5 (18.5)	7 (25.9)	7 (25.9)	5 (18.5)
*p* Value	0.54			0.79
OR (95% CI)	0.62 (0.13–2.89)		1.22 (0.26–5.66)	
MYCN				
Amplified	0	1 (3.7)	0	1(3.7)
Not amplified	13 (48.1)	13 (48.1)	15 (55.6)	11 (40.7)
*p* Value	NC		NC	
OR (95% CI)	NC		NC	
Relapse				
Yes	4 (14.8)	3 (11.1)	4 (14.8)	3 (11.1)
No	9 (33.3)	11 (40.7)	11 (40.7)	9 (33.3)
*p* Value	0.58		0.92	
OR (95% CI)	1.62 (0.28–9.25)		1.09 (0.19–6.19)	
Death				
Yes	1 (3.7)	5 (18.5)	2 (7.4)	4 (14.8)
No	12 (44.4)	9 (33.3)	13 (48.1)	8 (29.6)
*p* Value	0.04		0.22	
OR (95% CI)	6.66 (0.65–67.47)		3.25 (0.48–21.99)	

OR = unadjusted odds ratio; CI = confidence interval; NC = not calculable.

**Table 5:** The association between serum levels of anti-inflammatory cytokines stratified by low levels versus high levels and the clinicopathologic factors of NB patients.

	IL-10		TGF-β	
Variables	Low Levels	High Levels	Low Levels	High Levels
Risk				
Low/Intermediate	8 (29.6)	4 (14.8)	13 (48.1)	2 (7.4)
High	13 (48.1)	2 (7.4)	10 (37)	2 (7.4)
*p* Value	0.6		0.68	
OR (95% CI)	0.61 (0.09–3.82)		0.64 (0.074–5.41)	
INSS stage				
stage 1/2	4 (14.8)	3 (11.1)	5 (18.5)	2 (7.4)
stage 3/4	17 (63)	3 (11.1)	18 (66.6)	2 (7.4)
*p* Value	0.013		0.04	
OR (95% CI)	0.09 (0.01–0.6)		0.11 (0.01–0.97)	
Histology (Shimada)				
Favorable	11 (40.7)	4 (14.8)	11 (40.7)	4 (14.8)
Unfavorable	10 (37)	2 (7.4)	12 (44.4)	0
*p* Value	0.53		NC	
OR (95% CI)	1.81 (0.27–12.17)		NC	
MYCN				
Amplified	1 (3.7)	0	1 (3.7)	0
Not amplified	20 (74.1)	6 (22.2)	22 (81.5)	4 (14.8)
*p* Value	NC		NC	
OR (95% CI)	NC		NC	
Relapse				
Yes	6 (22.2)	1(3.7)	6 (22.2)	1(3.7)
No	15 (55.6)	5 (18.5)	17 (63)	3 (11.1)
*p* Value	0.56			0.96
OR (95% CI)	2 (0.19–20.89)		1.05 (0.09–12.23)	
Death				
Yes	4 (14.8)	2 (7.4)	6 (22.2)	0
No	17 (63)	4 (14.8)	17 (63)	4 (14.8)
*p* Value	0.46		NC	
OR (95% CI)	2.12 (0.28–15.96)		NC	

OR = unadjusted odds ratio; CI = confidence interval; NC = not calculable.

## Data Availability

The data presented in this study are available upon request from the corresponding author. The data are not publicly available due to the confidentiality of patient data.

## Appendix A

**Table A1:** Cut-off values for the cytokines evaluated.

Cytokine	Low Levels (pg/mL)	High Levels (pg/mL)
IL-1β	≤3.4	>3.4
IL-6	≤7	>7
IL-12 p40	≤41	>41
IL-12 p70	≤65	>65
TNF-α	≤10	>10
IFN-γ	≤20	>20
IL-8	≤40	>40
MCP-1	≤1000	>1000
IL-10	≤8	>8
TGF-β1	≤5000	>5000

**Table A2:** Characteristics of NB patients stratified by the serum levels of pro-inflammatory cytokines.

	Frequencies n (%) of Serum Levels of Pro-Inflammatory Cytokines.									
	IL-6		TNF-α		IL-12p40		IL-12p70		IFN-γ	
Variables	Low Levels	High Levels	Low Levels	High Levels	Low Levels	High Levels	Low Levels	High Levels	Low Levels	High Levels
INSS stage										
1	3 (11.1)	2 (7.4)	5 (18.5)	0	2 (7.4)	3 (11.1)	5 (18.5)	0	1 (3.7)	4 (14.8)
2b	2 (7.4)	0	2(7.4)	0	0	2 (7.4)	2 (7.4)	0	1 (3.7)	1 (3.7)
3	6 (22.2)	1 (3.7)	6 (22.2)	1 (3.7)	5 (18.5)	2 (7.4)	6 (22.2)	1 (3.7)	2 (7.4)	5 (18.5)
4	6 (22.2)	7 (25.9)	11 (40.7)	2 (7.4)	7 (25.9)	6 (22.2)	12 (44.4)	1 (3.7)	3 (11.1)	10 (37)
*p* Value	0.29		1		0.42		1		0.91	
INRG										
L1	4 (14.8)	1 (3.7)	5 (18.5)	0	3 (11.1)	2 (7.4)	5 (18.5)	0	2 (7.4)	3 (11.1)
L2	6 (22.2)	2 (7.4)	7(25.9)	1 (3.7)	4 (14.8)	4 (14.8)	7(25.9)	1 (3.7)	2 (7.4)	6 (22.2)
M	7 (25.9)	7 (25.9)	12 (44.4)	2 (7.4)	7 (25.9)	7 (25.9)	13 (48.1)	1 (3.7)	3 (11.1)	11 (40.7)
MS	0	0	0	0	0	0	0	0	0	0
*p* Value	0.36		1		1		1		0.84	
Risk										
Low	4 (14.8)	1 (3.7)	5 (18.5)	0	2 (7.4)	3 (11.1)	5 (18.5)	0	2 (7.4)	3 (11.1)
Intermediate	6 (22.2)	1 (3.7)	5 (18.5)	2 (7.4)	3 (11.1)	4 (14.8)	6 (22.2)	1 (3.7)	0	7 (25.9)
High	7 (25.9)	8 (29.6)	14 (51.9)	1 (3.7)	9 (33.3)	6 (22.2)	14 (51.9)	1 (3.7)	5 (18.5)	10 (37)
*p* Value	0.22		0.23		0.66		1		0.17	
Differentiation										
Undifferentiated	1 (3.7)	1 (3.7)	2 (7.4)	0	1 (3.7)	1 (3.7)	2 (7.4)	0	1(3.7)	1(3.7)
Partially differentiated	12 (44.4)	4 (14.8)	13 (48.1)	3 (11.1)	7 (25.9)	9 (33.3)	14 (51.9)	2 (7.4)	3 (11.1)	13 (48.1)
Differentiated	4 (14.8)	1 (3.7)	5 (18.5)	0	4 (14.8)	1 (3.7)	5 (18.5)	0	2 (7.4)	3 (11.1)
Not specified	0	4(14.8)	4 (14.8)	0	2 (7.4)	2 (7.4)	4 (14.8)	0	1(3.7)	3 (11.1)
*p* Value	0.022		0.79		0.67		1		0.67	
Histology										
Favorable	12 (44.4)	3 (11.1)	15 (55.6)	0	7 (25.9)	8 (29.6)	13 (48.1)	2 (7.4)	5 (18.5)	10 (37)
Unfavorable	5 (18.5)	7 (25.9)	9 (33.3)	3 (11.1)	7 (25.9)	5 (18.5)	12 (44.4)	0	2 (7.4)	10 (37)
*p* Value	0.05		0.07		0.41		0.29		0.29	
MYCN										
Amplified	0	1(3.7)	1 (3.7)	0	1 (3.7)	0	1 (3.7)	0	0	1 (3.7)
Not amplified	17 (63)	9 (33.3)	23 (85.2)	3 (11.1)	13 (48.1)	13 (48.1)	24 (88.9)	2 (7.4)	7 (25.9)	19 (70.4)
*p* Value	0.18		1		0.75		0.96		0.37	

*p* value obtained by X^2^ analysis and Fisher’s exact test, INSS = International Neuroblastoma Staging System, INRG = International Neuroblastoma Risk Group, MYCN = MYCN proto-oncogene.

**Table A3:** Characteristics of NB patients stratified by serum levels of chemokines.

	Frequencies n (%) of Serum Levels of Chemokines.			
	IL-8		MCP-1	
Variables	Low Levels	High Levels	Low Levels	High Levels
INSS stage				
1	1 (3.7)	4 (14.8)	2 (7.4)	3 (11.1)
2b	2 (7.4)	0	1 (3.7)	1 (3.7)
3	3 (11.1)	4 (14.8)	4 (14.8)	3 (11.1)
4	7 (25.9)	6 (22.2)	8 (29.6)	5 (18.5)
*p* Value	0.33		0.93	
INRG				
L1	3 (11.1)	2 (7.4)	3 (11.1)	2 (7.4)
L2	3 (11.1)	5 (18.5)	4 (14.8)	4 (14.8)
M	7 (25.9)	7 (25.9)	8 (29.6)	6 (22.2)
MS	0	0	0	0
*p* Value	0.77		1	
Risk				
Low	2 (7.4)	3 (11.1)	2 (7.4)	3 (11.1)
Intermediate	3 (11.1)	4 (14.8)	3 (11.1)	4 (14.8)
High	8 (29.6)	7 (25.9)	10 (37)	5 (18.5)
*p* Value	1		0.38	
Differentiation				
Undifferentiated	2 (7.4)	0	2 (7.4)	0
Partially differentiated	9 (33.3)	7 (25.9)	11 (40.7)	5 (18.5)
Differentiated	2 (7.4)	3 (11.1)	2 (7.4)	3 (11.1)
Not specified	0	4 (14.8)	0	4(14.8)
*p* Value	0.09		0.027	
Histology (Shimada)				
Favorable	8 (29.6)	7 (25.9)	8 (29.6)	7 (25.9)
Unfavorable	5 (18.5)	7 (25.9)	7 (25.9)	5 (18.5)
*p* Value	0.7		1	
MYCN				
Amplified	0	1 (3.7)	0	1(3.7)
Not amplified	13 (48.1)	13 (48.1)	15 (55.6)	11 (40.7)
*p* Value	0.25		0.22	

*p* Value obtained by X^2^ analysis and Fisher exact test, INSS = International Neuroblastoma Staging System, INRG = International Neuroblastoma Risk Group, MYCN = MYCN proto-oncogene.

**Table A4:** Characteristics of NB patients stratified by serum levels of anti-inflammatory cytokines.

	Frequencies n (%) of Serum Levels of Anti-Inflammatory Cytokines.			
	IL-10		TGF-β1	
Variables	Low Levels	High Levels	Low Levels	High Levels
INSS Stage				
1	3 (11.1)	2 (7.4)	4 (14.8)	1 (3.7)
2b	1 (3.7)	1 (3.7)	1 (3.7)	1 (3.7)
3	6 (22.2)	1 (3.7)	6 (22.2)	1 (3.7)
4	11 (40.7)	2 (7.4)	12 (44.4)	1 (3.7)
*p* Value	0.43		0.41	
INRG				
L1	4 (14.8)	1 (3.7)	4 (14.8)	1 (3.7)
L2	6 (22.2)	2 (7.4)	6 (22.2)	2 (7.4)
M	11 (40.7)	3(11.1)	13 (48.1)	1 (3.7)
MS	0	0	0	0
*p* Value	1		0.48	
Risk				
Low	3 (11.1)	2 (7.4)	4 (14.8)	1 (3.7)
Intermediate	5 (18.5)	2 (7.4)	6 (22.2)	1 (3.7)
High	13 (48.1)	2 (7.4)	13 (48.1)	2 (7.4)
*p* Value	0.47		1	
Differentiation				
Undifferentiated	1 (3.7)	1 (3.7)	2 (7.4)	0
Partially differentiated	14 (51.9)	2 (7.4)	13 (48.1)	3 (11.1)
Differentiated	3 (11.1)	2 (7.4)	5 (18.5)	0
Not specified	3 (11.1)	1 (3.7)	3 (11.1)	1 (3.7)
*p* Value	0.28		0.70	
Histology (Shimada)				
Favorable	11 (40.7)	4 (14.8)	11 (40.7)	4 (14.8)
Unfavorable	10 (37)	2 (7.4)	12 (44.4)	0
*p* Value	0.66		0.078	
MYCN				
Amplified	1 (3.7)	0	1 (3.7)	0
Not amplified	20 (74.1)	6 (22.2)	22 (81.5)	4 (14.8)
*p* Value	1		0.92	

*p* value obtained by X^2^ analysis and Fisher’s exact test, INSS = International Neuroblastoma Staging System, INRG = International Neuroblastoma Risk Group, MYCN = MYCN proto-oncogene.
